# Cyanobacteria from freshwater lakes in the Azores archipelago, Portugal: data from long term phytoplankton monitoring

**DOI:** 10.3897/BDJ.8.e51928

**Published:** 2020-06-09

**Authors:** Rúben Luz, Rita Cordeiro, Joana Vilaverde, Pedro M. Raposeiro, Amélia Fonseca, Vítor Gonçalves

**Affiliations:** 1 CIBIO, Centro de Investigação em Biodiversidade e Recursos Genéticos, InBIO Laboratório Associado, Pólo dos Açores, Ponta Delgada, Portugal CIBIO, Centro de Investigação em Biodiversidade e Recursos Genéticos, InBIO Laboratório Associado, Pólo dos Açores Ponta Delgada Portugal; 2 Faculdade de Ciências e Tecnologia, Universidade dos Açores, Ponta Delgada, Portugal Faculdade de Ciências e Tecnologia, Universidade dos Açores Ponta Delgada Portugal

**Keywords:** cyanobacteria, freshwater, oceanic islands, phytoplankton

## Abstract

**Background:**

The Azores are oceanic islands located in the Northern Atlantic Ocean and are particularly rich in aquatic systems, ranging from freshwater, brackish, marine and thermal habitats. Due to the increase in local anthropogenic pressures and global warming, several azorean lakes began to reveal signs of eutrophication that led to the implementation of monitoring programmes and management strategies on the most impacted lakes. Later, the Water Framework Directive (2000/60/EC) demanded the establishment of biomonitoring programmes for European freshwater ecosystems and the limited Azorean monitoring programmes were extended to a larger set of lakes. Since the establishment of the aquatic systems monitoring programme in the Azores archipelago, lakes have been regularly sampled, producing innumerous unpublished records of cyanobacteria that are difficult to access.

**New information:**

Here we present the occurrences of cyanobacteria in Azorean lakes that result from 22 years of phytoplankton monitoring in a total of 1948 cyanobacteria occurrences from 968 phytoplankton sampling events on Azorean lakes done between 1996 and 2018 as part of regional inland aquatic ecosystems monitoring programmes. Forty two cyanobacteria taxa were identified in those events, 28 species and 14 at genus level. This information is crucial for conservation, biodiversity studies and lake management, as some of the cyanobacteria species present are bloom-forming and have the ability to produce toxins. This will also allow for the identification of invasive species and possible targeted control and mitigation programmes, according to the species present in the Azorean lakes.

## Introduction

Cyanobacteria are common inhabitants of lake plankton from oligotrophic pristine environments to heavily-impacted eutrophic lakes (Whitton and Potts 2012). Although they can be very important in oligotrophic lakes, especially through unicellular small-size species (Stockner et al. 2007), they are especially dominant in eutrophic lakes, where filamentous and larger colonial species form frequent blooms that can cause surface scums and unpleasant odours (Reynolds and Petersen 2000). Furthermore, several bloom-forming cyanobacteria species produce toxins that can cause an impact on the ecosystem biodiversity, as well as on the safety of water resources for several uses (Watson et al. 2015). Several factors may contribute to the dominance of cyanobacteria in lakes, including higher temperature and lower TN/TP optima compared to other phytoplankton groups, low light-energy requirements, superior uptake kinetics for inorganic carbon, the ability to fix molecular nitrogen and buoyancy, amongst others (Dokulil and Teubner 2000).

In the last decades of the 20th century, several Azorean lakes started to show signs of eutrophication (Porteiro 2000), with increased cyanobacteria abundance and the occurrence of blooms (Santos et al. 2005). This caused generalised concern amongst local authorities and population and monitoring programmes to access lake trophic status and ecosystem health were implemented in the region.

The Azores are a nine-island archipelago in the middle of the North Atlantic Ocean, distant roughly 1500 km from Europe and 1900 km from North America, divided into three groups according to their geographical position. Aligned along 615 km in WNW-ESE trend, the western group includes Flores and Corvo islands, the central group being composed by Graciosa, Faial, Pico, São Jorge and Terceira islands, while the eastern group comprises Santa Maria and São Miguel islands. Despite their geographical proximity, islands have distinct features that distinguish them, such as their geological setting (Moore 1990, Azevedo and Portugal Ferreira 2006, Cole et al. 2008) and their age (Ávila et al. 2016).

The diversity of habitats amongst islands differs naturally due to their geological setting and origin, allowing a great diversity of inland habitats, such as freshwater lakes (Porteiro 2000, Cruz and França 2006, Morton 2014). According to Porteiro 2000, the Azores have 88 lakes, located in São Miguel, Terceira, Pico, São Jorge, Flores and Corvo islands. The lakes can be classified into two types, according to their origin: i) lakes within volcanic depressions and ii) lakes in topographically depressed areas (Constância et al. 2000, Antunes 2004). Azorean lakes presented great differences in their size, elevation and depth. The largest lake is in São Miguel island (358.7 ha - Lagoa Azul), while the small lakes < 1 ha are present in São Miguel, Pico, Terceira and São Jorge (Porteiro 2000). Most of the lakes are located between 600 and 800 m altitude, while the range is from 230 m (São Miguel island) and 1,050 m altitude (Pico island). Most of the lakes are shallow with maximum depths ranging from 0.4 to 7 metres. These lakes are permanently mixed and cyanobacteria rarely dominate their phytoplankton. Deeper lakes, with maximum depth between 14 and 115 m, have a monomictic warm thermal regime, presenting stratification normally between April and October. Contrary to shallow lakes, deep, stratified lakes, located at lower altitudes where human activities are more intense, have undergone eutrophication and their phytoplankton is dominated by cyanobacteria, especially during the stratification period.

Here we present detailed data on the distribution of species belonging to several groups of cyanobacteria in Azorean lakes from 22 years (1996-2018) of lake phytoplankton monitoring for trophic and ecological status assessment (Luz et al. 2020).

## Project description

### Title

Cyanobacteria from freshwater lakes in the Azores Archipelago, Portugal

### Personnel

Vítor Gonçalves, Rúben Luz

### Funding

This research was funded by Secretaria Regional do Ambiente – Governo dos Açores (Contrato Nº 8/2003/DROTRH), Secretaria Regional do Ambiente e do Mar – Governo dos Açores (Ajuste Direto Nº 18/2009; Concurso Público Nº 3/2009; Contrato Nº 3/2011), Secretaria Regional dos Recursos Naturais – Governo dos Açores (Concurso Público 1/DRA/2014), Fundo Regional para a Ciência e Tecnologia – Governo dos Açores (M3.1.a/F/017/2015) and by FEDER funds through the Interreg-MAC 2014-2020 Programme under the REBECA project - Red de excelencia en biotecnología azul (algas) de la región macaronesia (MAC1.1a/060). This work was also funded by Portuguese National Funds, through FCT − Fundação para a Ciência e a Tecnologia, the European Union, QREN, FEDER, COMPETE, by funding the CIBIO/InBIO (project UID/BIA/50027/2013 and POCI-01-0145-FEDER-006821). This manuscript is also a contribution to the updated checklist of Azorean cyanobacteria that is being prepared within the newly-launched project AZORESBIOPORTAL – PORBIOTA (ACORES-01-0145-FEDER-000072), financed by FEDER at 85% and by Azorean Public funds by 15% through Operational Programme Azores 2020.

## Sampling methods

### Study extent

Phytoplankton samples from 24 lakes in Corvo (1), Flores (6), Pico (5) and São Miguel (12) islands in the Azores archipelago were collected seasonally between 1996 and 2018.

### Sampling description

Phytoplankton samples were taken using a Van Dorn bottle and a 10 µm mesh plankton net at the lake's maximum depth point. In deep lakes (maximum depth > 10 m), until 2010, discrete samples were collected at surface, mid-water column depth and 1 m above the sediment. After 2010, a combined sample of the euphotic zone was obtained by mixing discrete 1 litre samples collected at 1 m intervals from the surface to the bottom of the euphotic zone. In shallow lakes (maximum depth < 10 m), a surface sample (integrated sample of the first metre of the water column) was collected. Net samples were obtained by a trawl from 1 m above the sediment to the water column surface. All samples were preserved with 1% Lugol solution (v/v) and taxa were identified to the lowest taxonomical level possible using light microscopy. For regular cyanobacteria identification and quantification, bottle samples were used, whereas net samples were used to detect rare or uncommon species that could be missed in the Van Dorn bottle samples. A final occurrence list was built by combining data from both samples. Preserved samples after analysis were deposited in Faculdade de Ciências e Tecnologia, Universidade dos Açores.

### Step description

Cyanobacteria were identified according to Komárek and Anagnostidis (1998), Komárek and Anagnostidis (2008) and Komárek (2013).

## Geographic coverage

### Description

**Figure 1.**
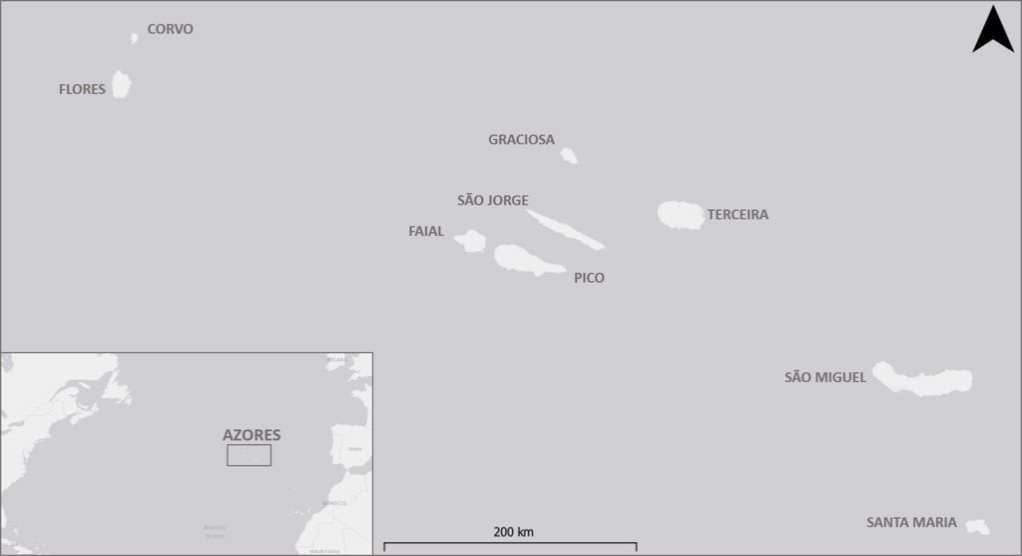
Azores archipelago position and island distribution.

Azores archipelago, Portugal (Fig. 1)

### Coordinates

36.9 and 39.8 Latitude; -31.5 and -24.9 Longitude.

## Taxonomic coverage

### Description

Cyanobacteria were identified to genus or species level. Nomenclature was updated to the most recent taxonomic treatment.

### Taxa included

**Table:** 

Rank	Scientific Name	Common Name
class	Cyanophyceae Schaffner	Cyanophyceae

## Traits coverage

### Data coverage of traits

PLEASE FILL IN TRAIT INFORMATION HERE

## Temporal coverage

### Notes

1996-12-16 through 2018-06-21

## Usage rights

### Use license

Other

### IP rights notes

This work is licensed under a Creative Commons Attribution (CC-BY) 4.0 Licence.

## Data resources

### Data package title

Cyanobacteria from freshwater lakes in the Azores Archipelago, Portugal

### Resource link

http://ipt.gbif.pt/ipt/resource?r=cyanobacteria_ocurrence

### Alternative identifiers

https://www.gbif.org/dataset/9d9d16b2-b6ad-433f-9baa-5354132896ac

### Number of data sets

2

### Data set 1.

#### Data set name

Cyanobacteria from freshwater lakes in the Azores Archipelago, Portugal - event

#### Data format

Darwin Core

#### Number of columns

18

#### Description

Cyanobacteria sampling events in lakes from the Azores Archipelago, based on data from monitoring programmes dated from 1996 to 2018.

**Data set 1.** 

Column label	Column description
id	Unique Identifier
type	The nature of the resource
eventID	Identifier unique for the dataset
parentEventID	Identifier of the sampling campaign, unique for the dataset
samplingProtocol	The sampling method used
eventDate	Date when the event occurred
habitat	The habitat type in which the event occurred
continent	Continent body in which sampling location occurs
waterBody	Water body in which sampling location occurs
islandGroup	Island group in which sampling location occurs
island	Island in which sampling location occurs
country	Country in which sampling location occurs
countryCode	Country Code in which sampling location occurs
municipality	Municipality in which sampling location occurs
locality	The specific description of the place in which sampling occurs
decimalLatitude	The geographic latitude, in decimal degrees
decimalLongitude	The geographic longitude, in decimal degrees
geodeticDatum	The reference point for the various coordinate systems used in mapping the earth

### Data set 2.

#### Data set name

Cyanobacteria from freshwater lakes in the Azores Archipelago, Portugal - occurrence

#### Data format

Darwin Core

#### Number of columns

12

#### Description

Cyanobacteria occurrence in lakes from the Azores Archipelago, based on data from monitoring programmes dated from 1996 to 2018.

**Data set 2.** 

Column label	Column description
id	Identifier
type	The nature of the resource
basisOfRecord	The nature of the data record
occurrenceID	Identifier of the occurrence, coded as a global unique identifier
eventID	Identifier of the event from where the occurrence is based
identifiedBy	The person responsible for assigning the taxon to the subject
scientificName	The full scientific name including author
acceptedNameUsage	The full scientific name including author currently accepted
kingdom	Kingdom name in which the taxon is classified
phylum	Phylum name in which the taxon is classified
class	Class name in which the taxon is classified
taxonRank	Lowest taxonomic rank of the taxon
